# Impulsive prepotent actions and tics in Tourette disorder underpinned by a common neural network

**DOI:** 10.1038/s41380-020-00890-5

**Published:** 2020-09-29

**Authors:** Cyril Atkinson-Clement, Camille-Albane Porte, Astrid de Liege, Yanica Klein, Cecile Delorme, Benoit Beranger, Romain Valabregue, Cecile Gallea, Trevor W. Robbins, Andreas Hartmann, Yulia Worbe

**Affiliations:** 1 Sorbonne University, 75005 Paris; Inserm U1127, CNRS UMR7225, UM75, ICM, F-75013 Paris, France; 2 Movement Investigation and Therapeutics Team, ICM, Paris, France; 3 National Reference Center for Tourette syndrome, Assistance Publique-Hôpitaux de Paris, Groupe Hospitalier Pitié-Salpêtrière, F-75013 Paris, France; 4 Centre de NeuroImagerie de Recherche (CENIR), Sorbonne Université, UMRS975, CNRS UMR7225, ICM, F-75013 Paris, France; 5 Behavioural and Clinical Neuroscience Institute, University of Cambridge, Cambridge, UK; 6 Department of Psychology, University of Cambridge, Cambridge, UK; 7 Department of Neurophysiology, Saint Antoine Hospital, Assistance Publique-Hôpitaux de Paris, Paris, France

**Keywords:** Psychiatric disorders, Neuroscience

## Abstract

Tourette disorder (TD), which is characterized by motor and vocal tics, is not in general considered as a product of impulsivity, despite a frequent association with attention deficit hyperactivity disorder and impulse control disorders. It is unclear which type of impulsivity, if any, is intrinsically related to TD and specifically to the severity of tics. The waiting type of motor impulsivity, defined as the difficulty to withhold a specific action, shares some common features with tics. In a large group of adult TD patients compared to healthy controls, we assessed waiting motor impulsivity using a behavioral task, as well as structural and functional underpinnings of waiting impulsivity and tics using multi-modal neuroimaging protocol. We found that unmedicated TD patients showed increased waiting impulsivity compared to controls, which was independent of comorbid conditions, but correlated with the severity of tics. Tic severity did not account directly for waiting impulsivity, but this effect was mediated by connectivity between the right orbito-frontal cortex with caudate nucleus bilaterally. Waiting impulsivity in unmedicated patients with TD also correlated with a higher gray matter signal in deep limbic structures, as well as connectivity with cortical and with cerebellar regions on a functional level. Neither behavioral performance nor structural or functional correlates were related to a psychometric measure of impulsivity or impulsive behaviors in general. Overall, the results suggest that waiting impulsivity in TD was related to tic severity, to functional connectivity of orbito-frontal cortex with caudate nucleus and to structural changes within limbic areas.

## Introduction

Tourette disorder (TD) is characterized by motor and vocal tics and is frequently associated with comorbid disorders related to impulsivity, such as attention deficit hyperactivity disorder (ADHD), obsessive-compulsive disorder (OCD), and impulse control disorders (ICD [1–3]). Impulsivity is a broad and multifaceted concept, that has been suggested to have different forms, e.g., impulsive action vs. impulsive choice and “waiting” vs. “stopping” impulsivity, which are also supported by distinct behavioral and neural mechanisms [4].

In TD, the possible overlap between impulsivity and tics remains a subject of debate [1, 3, 5] since it could be intrinsically related to the disorder or could result from comorbidities or antipsychotic treatment. As tics, the hallmark of TD, are sometimes considered as semi-voluntary actions, previous studies have focused on motor impulsivity, especially on stopping impulsivity, defined as the capacity for inhibition, cancellation, or “braking” of initiated actions. This type of impulsivity is usually assessed by using the stop signal reaction time test (inhibition of on-going action) and the go-no go test (inhibition of actions in preparation), and has shown discrepant results in patients with TD which potentially were confounded by antipsychotic treatments and comorbidities [6, 7].

Waiting impulsivity represents another form of motor impulsivity and can be described as the inability to withhold a specific action until the explicit action cue is provided: to date, it has received little to no attention in TD. In humans and animals, waiting impulsivity can be measured using 4-choice serial reaction time tasks (4CSRTT) [8, 9], where premature responding, i.e., responses occurring prior to cue presentation, provides an objective index of impulsive behavior.

Premature responding and tics have some features in common. First, waiting impulsivity has been related to an excess of dopaminergic neurotransmission within the striatum in animal models [4], which has also been recognized as one of pathophysiological mechanism of tics in TD [10]. Moreover, the neural network including ventromedial prefrontal, anterior cingulate, insular cortices, hippocampus, subthalamic nucleus, and both dorsal and ventral striatum was shown to mediate premature responses in the 4CSRTT [4, 11]. Structural or functional abnormalities of most of these regions have also been identified in TD [12, 13]. Finally, some studies suggested abnormal interactions between limbic and motor networks as pathophysiological mechanisms underpinning tics [14–16].

The first aim of this study was to evaluate the relationship between waiting impulsivity and TD, hypothesizing that TD patients would show a propensity to impulsive action and a greater number of premature responses on the 4CSRTT. We also aimed to study the functional and structural brain changes related to waiting impulsivity in TD.

## Materials and methods

### Subjects

The study was approved by the ethics committee (CCP16163/C16-07) and preregistered prior to the research being conducted on ClinicalTrial (clinicaltrials.gov/ct2/show/NCT02960698). All subjects gave informed consent. We recruited adult TD patients and sex-, educational level and age-matched healthy controls (HC). For all participants, the exclusion criteria were: incapacity or unwillingness to give consent for the study; substance addiction (excluding nicotine and recreational use of cannabis, i.e., less than once per week); history of psychosis and presence of neurological (including childhood tics) or psychiatric conditions for HC.

All participants were screened for the presence of psychiatric disorders (Mini International Neuropsychiatric Interview, MINI [17]), ICD (Minnesota Impulse Disorders Interview, MIDI [18]), impulsivity (Barratt Impulsivity Scale, BIS-11 [19]) and anxiety (State-Trait Anxiety Inventory, STAI [20]). Tic severity was assessed using the Yale Global Tic Severity Scale (YGTSS [21]) and the presence of psychiatric comorbidities was also evaluated from medical records and psychiatric evaluation prior to inclusion in the study.

### Four choice serial reaction time task

**Fig. 1:**
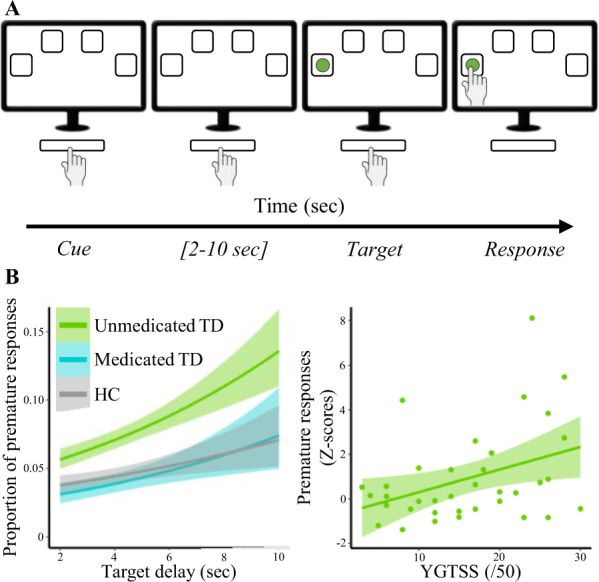
**a** Four Choice Reaction Time Task paradigm; (**b**) Results of generalized linear mixed model showing an increased proportion of premature responses in group of unmedicated TD patients (left), (right) correlation of premature responses with severity of tics (YGTSS/50). HC healthy controls, TD Tourette disorder patients, YGTSS Yale Global Tic Severity Scale.

Waiting impulsivity was assessed with the 4CSRTT (Fig. 1a) and was programmed with Visual Basic for a total duration of 30 min [9].

Participants were positioned in front of a touch-screen computer and were instructed to press and hold down the space bar with the dominant index finger. A press of the space bar indicated ‘cue onset’ and 4 boxes appeared on the screen. After a random cue-target interval (2–10 s), a target (green circle) appeared during 32–64 msec in one of the boxes. Participants were asked to respond as quickly as possible by releasing the space bar and by touching the corresponding box on the screen. The task included 2 baseline blocks (20 trials per block) without monetary feedback and 4 test blocks (40 trials per block) with monetary feedback. To increase premature responding (i.e., release the space bar before the cue-target presentation), the testing blocks included decreasing and increasing target time, variable cue-target intervals, and the introduction of a distractor.

For each participant, mean and standard deviations (SD) of reaction time (RT) during each baseline block were used to set individualized feedback in the test blocks. The subjects could earn 1€ (less than mean RT-(0.5 × SD)), 0.5€ (mean RT ± (0.5 × SD)), 0.1€ (less than mean RT + (1.5 × SD)) or lose 1€ (more than mean RT + (1.5 × SD)). If participants made no response, the feedback was a loss of 0.5€. Following a premature response, no reward was earned. The total won was also specified on the feedback display.

### Neuroimaging data acquisition and pre-processing

All subjects were enrolled in a multi-modal MRI protocol on a 3 T Siemens Scanner (PRISMA) with a 64-channel head coil.

The MRI sequences parameters were as follows: (i) a sagittal T1-weighted magnetization prepared rapid gradient echo (MP2RAGE) sequence (TR = 5 s, TI = 700/2500 msec, fov = 256, 1 mm isotropic, Ipat acceleration of 3), (ii) a multi-echo echo-planar imaging (EPI) sequence of 11 min duration for the resting-state, with a multi-slice, multi-echo acquisition scheme (TR = 1.9 s, TE = 17/36/56 msec, Ipat acceleration factor 2, Multi-band 2, isotropic voxel size 3 mm, dimensions = 66 × 66 in plane × 46 slices), acquired with eyes opened and fixed on the cross which was monitored by an eye tracker; (iii) a multi-shell diffusion tensor imaging (DTI) acquisition (TR = 3.5 s, TE = 75 msec, Multi-band of 3, isotropic voxel size = 1.75 mm, 60 direction with *b* = 2000 s/mm^2^, 32 direction with *b* = 1000 s/mm^2^, 8 direction with *b* = 300 s/mm^2^, one *b* = 0 was each 10 directions). For each EPI sequence (resting-state fMRI and DTI) one extra volume of opposite phase direction was acquired.

T1-weighted images (MP2RAGE) were first denoised using a Matlab implementation of the algorithm (www.github.com/JosePMarques/MP2RAGE-related-scripts [22]) and pre-processed (segmentation into gray and white matter and cerebrospinal fluid, normalization to MNI space, smoothing 10 mm) using the Computational Anatomy Toolbox (www.neuro.uni-jena.de/cat/) extension from SPM12 (www.fil.ion.ucl.ac.uk/spm/).

Functional MRI multi-echo data were processed with the open-source MEICA toolbox (www.github.com/ME-ICA/me-ica/), in version v3.2 beta1. This toolbox implements specific pre-processing steps for multi-echo data, then uses the different echos to perform decomposition in spatial and temporal maps in order to separate blood oxygen level dependent (BOLD) components from non-BOLD components in the signal. All processing steps were detailed in [23, 24]. Briefly, all echos were slice-time corrected and realigned to the first TR using rigid-body motion corrected, where registration was driven by the first echo and applied to all other echos. Echos were then aligned to the anatomical volume using affine registration, and a single warp was performed to combine all pre-processing steps. The final step included in MEICA was the decomposition and denoising of the weighted average of all echo-times using a principal component analysis to reduce dimensionality of the dataset by removing thermal noise followed by an independent component analysis to separate BOLD (blood-oxygen-level dependent) from non-BOLD components. The main output of MEICA was the denoised time-series that only contained BOLD signal and low variance components. The residual movements were then assessed (reported in Supplementary materials) and compared between the groups of patients as a part of the quality control.

DTI data (fractional anisotropy [FA] and mean diffusivity [MD]) pre-processing was performed using the FMRIB software library pipeline, including correction for motion and eddy currents, removal of nonbrain tissue of each volumes, tensor reconstruction, nonlinear registration, alignment to the MNI space and thresholding data at 0.2.

### Neuroimaging data analysis

#### fMRI

Functional connectivity analysis was performed using DPSARF toolbox (http://rfmri.org/DPARSF) and 122 regions from the AAL atlas were used for the construction of whole brain correlation matrix. We performed one-sample *t* tests to select resting-state BOLD signal correlations significantly different from 0, followed by a regression analysis with z-transformed premature responses. For both tests, a significance level was set at *p* ≤ 0.01 following permutation adjustments (*n* = 5000).

#### MP2RAGE and DTI

We performed whole-brain multivariate voxel-based regression analyses using the Permutation Analysis of Linear Models implemented in Matlab [25], on gray matter signal and the DTI metrics (FA and MD) including the total intracranial volume as covariable of noninterest and the *z*-score of premature response as covariable of interest. The threshold for significance was set at *p* ≤ 0.05 following familywise error correction corrected within modality and within contrast, following probabilistic threshold-free cluster enhancement [26] and 5000 permutations.

The signal from statistically significant clusters was extracted using the Marsbar toolbox [27]. We performed bootstrapped correlations of the signal with clinical measures as well as groups comparisons using Anova with Tukey correction for multiple comparisons post-hoc.

### Statistical analysis of clinical and behavioral data

All behavioral analyses were performed with R software. Demographic data were analyzed using *t* tests or chi-square analysis when appropriate. For the 4CSRTT, we excluded outliers data based on response time (inferior to 150 ms and superior to the general mean + [2 × SD]). Training blocks were not included in final analysis. Behavioral data of the 4CSRTT were analyzed using generalized mixed models and Tukey post-hoc for significant main effects. We considered group effects (TD, HC) and included other behavioral comorbidities (presence of ADHD and OCD) as well as medication with antipsychotic drugs (with or without medication; details on medication is provided in Supplementary Table 1). We analyzed the proportion of premature responses and the response time using mixed models with subjects and trial number as random effects. We performed Pearson’s correlations among the significant effects and clinical data. The threshold for significance for all tests was set at *p* ≤ 0.05. Lastly, mediation analyses were performed to determine the effect of a variable to another after an adjustment by a third variable (mediator).

## Results

### Subjects’ clinical and demographic characteristics

We recruited 64 TD patients and 34 HC. After quality inspection, noncomplete data on 9 TD and 3 HC (one or more modalities of MRI missing or low quality, all subjects performed the task) were excluded from the final analysis.

As shown in Table 1, there were no significant demographic differences between HC and TD groups. TD patients showed a higher level of anxiety (STAI), impulsivity (BIS-11), and number of expressed impulsive behaviors (MIDI). Sub-groups of TD patients either medicated with antipsychotics (*n* = 19) or medication free (unmedicated TD group, *n* = 36) had similar demographics and clinical scores, except for anxiety which was higher in unmedicated TD (F_(1;53)_ = 9.27, *p* = 0.004).

**Table 1:** Summary of demographics and clinical data.

	HC	TD (all)	TD (medicated)	TD (unmedicated)	HC vs. TD (all)	HC vs. TD medicated vs. TD unmedicated
Number of participants	31	55	19	36	–	–
Gender (M/F)	22/9	44/11	14/5	30/6	0.49	0.46
Age (y)	31.2 ± 10.5	29.8 ± 10.5	31 ± 9.4	29.1 ± 11.1	0.53	0.68
Years of education	14.5 ± 2.9	14.1 ± 2.6	14 ± 2.9	14.2 ± 2.4	0.52	0.77
MIDI	0.3 ± 0.7	1.5 ± 1.3	1.5 ± 1.3^a^	1.5 ± 1.3^a^	<0.001	<0.001
BIS-11	58.7 ± 9.7	65.1 ± 10.8	66.6 ± 12.3^a^	64.2 ± 10	0.007	0.02
STAI	62.3 ± 14.6	79.9 ± 18.6	89.7 ± 17.1^a,b^	74.7 ± 17.5^b^	<0.001	<0.001
YGTSS (/50)	–	16.4 ± 7.2	17.1 ± 5.8	16.1 ± 7.8	–	0.61
ADHD	–	6	3	3	–	0.88
OCD	–	3	1	2	–	0.99
IEO	–	6	4	2	–	0.76
ADHD + OCD	–	2	0	2	–	0.81
ADHD + IEO	–	13	2	11	–	0.75
OCD + IEO	–	3	1	2	–	0.99
ADHD + OCD + IEO	–	4	1	3	–	0.93

*ADHD* attention deficit hyperactivity disorder, *BIS-11*: Barratt impulsivity scale, *F* female, *HC* healthy controls, *IEO* intermittent explosive outbursts, *M* male, *MIDI* Minnesota impulse disorders interview, *OCD* obsessive-compulsive disorder, *STAI* state-trait anxiety inventory, TD Tourette disorder patients, YGTSS Yale Global Tic Severity Scale.

^a^Significant differences with HC following Tukey post-hoc.

^b^Significant differences between medicated and unmedicated TD patients following Tukey post-hoc.

### Four choice serial reaction time task performance

For the 4CSRTT (Fig. 1b), mixed models showed an increase in premature responding with increasing delay of target presentation (*F*_(1;13772)_ = 76.73, *p* < 0.0001), which was associated with antipsychotic medication (*F*_(2;13772)_ = 3.53, *p* = 0.028) but not with the life-time diagnosis of ADHD, OCD or intermittent explosive disorders (IEO) (all *p* > 0.05). Unmedicated TD showed more premature responses compared to HC (OR = 1.72, *p* = 0.04) and with a trend compared with medicated TD (OR = 1.75, *p* = 0.078). There was no difference between medicated TD and HC (OR = 0.98, *p* = 0.99). In addition, we found that RTs were longer in trials with delayed target presentation (*F*_(1;13780)_ = 156.07, *p* < 0.0001), but were not influenced by antipsychotic medication, gender, ADHD, OCD, or IEO (all *p* > 0.05).

Z*-*score of premature responses correlated with YGTSS/50 only in unmedicated TD (*r* = 0.375 with 95HDI = [0.061;0.629]), but not with other measures in either group of patients.

### Functional correlates of behavioral performance in unmedicated TD

We considered only variables that showed significant behavioral results, namely the z-score of premature responses in unmedicated TD.

**Fig. 2:**
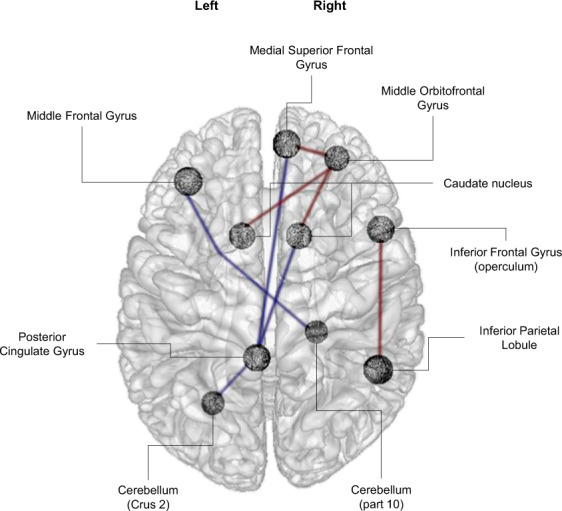
Functional connectivity results. Functional connectivity correlated with premature responses (*z*-score transformation) in unmedicated TD (red for positive correlations and blue for negative correlations).

As shown in Fig. 2, analysis showed that higher z-scored premature responses correlated with higher connectivity between (i) the right orbitofrontal cortex with the caudate nucleus (bilaterally) and the right medial superior frontal gyrus, and (ii) the right inferior frontal cortex (operculum) with the right inferior parietal cortex.

Higher z-scored premature responses correlated with lower connectivity between (i) the posterior part of the left cingulate gyrus with the right caudate nucleus, the medial superior frontal cortex (right) and the cerebellum (crus 2 left), and (ii) the cerebellum (lobule 10 right) with the middle frontal gyrus (left). There were no significant differences in these connections among the groups.

**Fig. 3:**
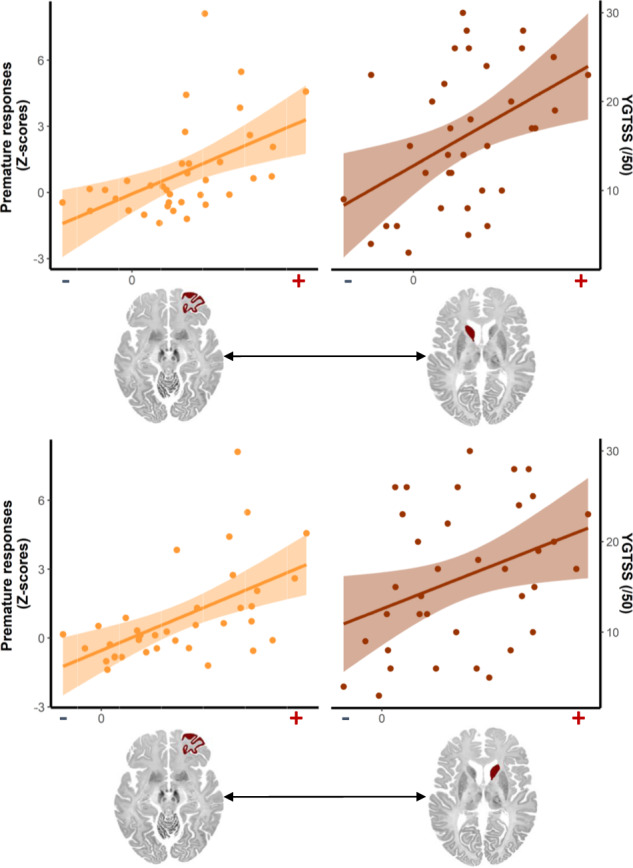
Premature response correlation with functional connectivity and severity of tics. Dual correlations involving the connectivity between the middle orbitofrontal gyrus and the left (top panel) and right (bottom panel) caudate nucleus with premature responses (*z*-score transformation, right correlations) and the YGTSS/50 (left correlations) for unmedicated TD. YGTSS: Yale Global Tic Severity Scale.

Connectivity between the right middle orbitofrontal cortex and the right (*F*_(1;34)_ = 5.04, *r* = 0.359, *p* = 0.031) and the left (*F*_(1;34)_ = 8.64, *r* = 0.45, *p* = 0.006) caudate nucleus positively correlated with the YGTSS/50 (Fig. 3). No significant correlations were found with the BIS-11 and the MIDI scale results.

To further investigate the possible relation between tic severity and premature responses, we used mediation analyses including the effect of YGTSS/50 on the z-scored premature responses and connectivity between the right orbitofrontal and the right/left caudate nucleus as mediators. Tic severity did not account directly for premature responses (right caudate: *p* = 0.159; left caudate: *p* = 0.233) but this effect was mediated by connectivity between the right orbitofrontal cortex and the right (*p* = 0.014) and left (*p* = 0.014) caudate nucleus.

### Structural correlates of behavioral performance in unmedicated TD

**Fig. 4:**
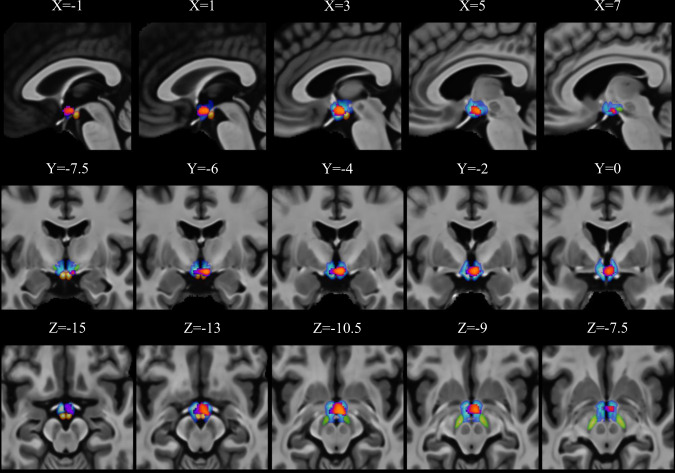
Anatomical correlates of premature response z-scores in group of unmedicated TD patients. Purple to red overlay represents the significant cluster. Blue, green and red overlays represents respectively the hypothalamus, the subthalamic nucleus, and the mamillary bodies (based on the Pauli subcortical atlas [60]).

As shown in Fig. 4, we found that the z-scored premature responses in unmedicated TD correlated with increased gray matter signal in one cluster (*x* = 3, *y* = −4.5, *z* = −12, *k* = 234, *t* = 1207.73) composed by the mammillary bodies and the hypothalamus bilaterally, and a part of the right antero-medial limbic part of subthalamic nucleus. No significant results were found for DTI metrics.

Following signal extraction of this cluster and group comparison (*F*_(2;83)_ = 3.17; *p* = 0.047), we found that unmedicated TD had a higher signal compared to HC (*p* = 0.038). There was no difference between HC and medicated TD as well as between the two patients’ groups. Correlational analyses of signal from the cluster showed no significant results with YGTSS/50, BIS-11, MIDI, or STAI.

## Discussion

Using the 4CSRTT to assess waiting impulsivity, we showed that unmedicated TD had a higher number of premature responses than HC, that was independent of ADHD and OCD, but correlated with tics severity. The relationship between tic severity and premature responses was mediated by functional connectivity of the right orbito-frontal cortex with the caudate nucleus bilaterally. Neither behavioral performance nor structural or functional correlates were related to a psychometric measure of impulsivity such as the BIS-11 or impulsive behaviors in general as indexed by MIDI, suggesting an exclusive relationship between waiting impulsivity and tic severity. In addition, the propensity to impulsive action in unmedicated TD was related to (i) a higher gray matter signal in the limbic structures (the mammillary bodies, the hypothalamus and a limbic part of the subthalamic nucleus) and (ii) connectivity of posterior cingulate and medial frontal gyrus with the basal ganglia and cerebellar regions at a functional level.

Consistent with our results, previous studies addressed the question of control of prepotent actions in TD using the Simon task, where subjects perform a motor response to stimuli presented in congruent (same side) and incongruent trials (different side, with a natural tendency to respond toward the source of stimulation) and showed a deficit in adult TD patients on this task, but no correlation of the performance with tics severity [28]. Functional MRI of adult TD patients performing the Simon task showed hyperactivity of the prefrontal cortex and the anterior cingulate cortex, as well as the caudate and pallidum, and a positive correlation of prefrontal cortex activity in this task with tics severity [29]. In children with TD, this deficit in control of prepotent actions during this task was related to the presence of ADHD and was improved by the prospect of reward [30].

However, and in contrast to the 4CSRTT, the Simon task requires suppression of impending actions, which occurs in the response-selection stage, rather than in waiting for a response cue. Moreover, these two tasks are also supported by different neural networks: the Simon task is underpinned by activity of the frontoparietal network and supplementary motor area [31], whereas the 4CSRTT is mostly dependent on limbic brain structures [4]. Consistently with these previous reports on the anatomy of waiting impulsivity, we found that the premature response in unmedicated TD patients was underpinned by a higher gray matter signal in limbic structures, including the mammillary bodies, the hypothalamus and a limbic part of the subthalamic nucleus. These structures are involved with different aspects of waiting impulsivity. For instance, lesions to [32] or manipulation of the activity of the subthalamic nucleus using deep brain stimulation [33] results in an increase of premature responding in this task. The hypothalamus was also shown to be implicated in impulsive actions via its connections with the hippocampus [34] as well as with the mesolimbic dopaminergic system [35]. The mammillary bodies were shown to be implicated in the anticipation of reward in humans [36]. In mice, pharmacological manipulation by picrotoxin injections in the area of mammillary bodies resulted in activation of brain structures associated with motivational processes, and facilitated locomotion [37]. The exact nature of a higher gray matter signal in these structures in TD patients is unknown but could reflect either a higher structural volume or a higher cellular and/or fiber density.

Both premature responses and tic severity positively correlated with each other and, at the neural level, with functional connectivity of the orbitofrontal cortex and the caudate nuclei. Mediation analysis showed that the orbitofrontal cortex-caudate nucleus functional connectivity mediated the relationship between tic severity and premature responding. Previous studies on humans and animals have pointed to a relationship between waiting impulsivity and orbitofrontal-striatal networks [38]. Neuronal recording in rodents during this task suggested that accumulation of neuronal activity in the medial orbitofrontal cortex was related to the delay in trials and was terminated by either accurate or premature response execution [39]. Optogenetic manipulation of the orbitofrontal cortex–striatal pathway was shown to modulate impulsivity related to delayed rewards [40].

In TD, tics have been primarily related to dysfunction of the sensori-motor pathways [12, 13] but recent studies in pediatric TD have pointed to abnormal structure of white matter underlying the orbitofrontal cortex bilaterally [41]. Earlier studies also suggested smaller caudate volumes in TD patients compared to controls [42]. In healthy volunteers, during a functional MRI study with a habit formation task, the orbitofrontal cortex and caudate nucleus showed higher activity on trials demanding cognitive control over prepotent habitual responses [43]. Interestingly, unmedicated TD patients showed a greater proportion of habitual response in this task, supported by the structure of sensorimotor networks [44]. Some studies have pointed to enhanced cognitive control in TD patients, but further work is needed to unravel the mechanisms of cognitive control over prepotent actions.

Waiting impulsivity also correlated with a lower functional connectivity of the posterior part of the left cingulate gyrus with the right caudate nucleus, the medial superior frontal cortex (right) and with the cerebellum (crus 2), and the middle frontal gyrus with the cerebellum (lobule 10).

The posterior cingulate cortex has been suggested to be a key region that links distinct functional networks to enable efficient cognitive function, in particular, working memory and focused attention [45]. One fMRI study that addressed the functional correlates of waiting impulsivity in HC showed the discriminative activity of middle frontal gyrus between subjects with high and low waiting impulsivity [46]. Further large-scale studies will be warranted to address the potential role of clinical and neurobiological heterogeneity of TD on task performance and network dysfunction in light of recent findings suggesting that brain networks are differentially altered in adults and children with TD [47].

In contrast to unmedicated TD, the performance of the medicated TD group did not differ from HC on the 4CSRTT. This result suggested that conventional antipsychotic treatment, mostly aripiprazole in our group, could both effectively reduce tics [48, 49] and modify the propensity to premature response. Aripiprazole, a D2 receptor antagonist, also has both an agonistic (5-HT1A and 5-HT2C) and an antagonistic (5-HT2A, 5-HT2B, and 5-HT7) effect at several serotonin receptors [50] and as a partial agonist at D2 dopamine receptors [51], acts to stabilize brain dopaminergic activity [52]. As TD is putatively related to a hyper-dopaminergic state, the stabilizing effect of aripiprazole on dopaminergic transmission could explain our findings. Alternatively, and consistent with decreased waiting impulsivity in medicated TD and the pharmacological profile of aripiprazole, rodent studies have reported that waiting impulsivity can be reduced by administration of 5-HT1A/B and 5-HT2C agonists [53, 54] and 5-HT2A antagonists [55–57] as well as by D2 receptor agonists and antagonists [58, 59].

### Limitations

The MRI findings are only correlational with behavioral measures of waiting impulsivity, so the possible causal role of specific anatomical networks cannot be demonstrated. Studies using functional imaging during the task would be warranted to confirm the implication of the brain networks identified in this study in waiting impulsivity in TD patients. Second, we are unable to firmly conclude about aripiprazole’s ameliorative effect on waiting impulsivity in TD patients, as we have not compared the same patient On and Off medication. A prospective within-subject study would be warranted to answer the question on the effect of aripiprazole on this waiting impulsivity. However, these issues do not preclude the main conclusions from our study that waiting impulsivity is selectively enhanced in unmedicated TD patients and associated with changes in defined neuroanatomical networks.

## Supplementary information

Supplementary Materials
