# Supplementary material for: Impulsive prepotent actions and tics in Tourette disorder underpinned by a common neural network
Source: Mol Psychiatry. 2020 Sep 29;26(7):3548–57. doi: 10.1038/s41380-020-00890-5 (PMC8505252; doi:10.1038/s41380-020-00890-5)
Supplement: Supplementary file 1 — Supplementary Materials [file 41380_2020_890_MOESM1_ESM.doc]

**Supplementary materials**

To control for movements artefacts, we performed statistical analyses on movements metrics after functional MRI pre-processing and found no group (HC vs. TD) difference for the mean framewise displacement (p=0.92), the standard deviation of the framewise displacement (p=0.94), the maximal value of framewise displacement (p=0.95) and the root mean square values (p=0.97).

In addition, no significant correlation was found between movements metrics and 4CSRTT z-scores proportion of premature response, for all TD patients (p>0.66) and for unmedicated TD (p>0.57).

**Supplementary Table. Medication of the medicated TD patients.**

| **Subject** | **Third generation neuroleptics** | **Second generation neuroleptics** | **Typical neuroleptics** | **Others** |
| --- | --- | --- | --- | --- |
| **1** |  |  | Pimozide (0.25mg/d) |  |
| **2** | Aripiprazole (2.5mg/d) |  |  |  |
| **3** | Aripiprazole (5mg/d) |  |  |  |
| **4** | Aripiprazole (5mg/d) |  |  |  |
| **5** |  | Risperidone (2mg/d) |  | Topiramate (100mg/d) |
| **6** | Aripiprazole (10mg/d) |  |  |  |
| **7** | Aripiprazole (5mg/d) |  |  |  |
| **8** | Aripiprazole (5mg/d) |  |  |  |
| **9** | Aripiprazole (2.5mg/d) |  |  |  |
| **10** | Aripiprazole (5mg/d) |  |  |  |
| **11** | Aripiprazole (5mg/d) |  |  | Fluoxetine (20mg/d) |
| **12** | Aripiprazole (5mg/d) |  |  |  |
| **13** | Aripiprazole (1.25mg/d) |  |  |  |
| **14** |  |  | Haloperidol (0.5mg/d) | Escitalopram (10mg/d) |
| **15** | Aripiprazole (5mg/d) |  |  |  |
| **16** | Aripiprazole (15mg/d) |  |  | Mianserin (60mg/d)  Fluoxetine (40mg/d)  Prazepam (10mg/d) |
| **17** | Aripiprazole (5mg/d) |  |  |  |
| **18** |  | Risperidone (1mg/d) |  |  |
| **19** | Aripiprazole (10mg/d) |  |  |  |
